# LncRNA-COX2 inhibits Fibroblast Activation and Epidural Fibrosis by Targeting EGR1

**DOI:** 10.7150/ijbs.67974

**Published:** 2022-01-16

**Authors:** Lei Yang, Shengnai Zheng, Dawei Ge, Mingjie Xia, Haijun Li, Jian Tang

**Affiliations:** 1 Taizhou Clinical Medical School of Nanjing Medical University, Taizhou People's Hospital, Taizhou, Jiangsu 225300, China.; 2 Department of Orthopedics, Hospital Affiliated 5 to Nantong University, Taizhou, Jiangsu 225300, China.; 3 School of Biomedical Engineering and Informatics, Nanjing Medical University, Nanjing, Jiangsu 211166, China.; 4 Department of Orthopedics, Nanjing First Hospital, Nanjing Medical University, Nanjing, Jiangsu 210006, China.; 5 Department of Plastic and Burn Surgery, the First Affiliated Hospital of Nanjing Medical University, Nanjing, Jiangsu 210029, China.

**Keywords:** LncRNA-COX2, Fibroblast Activation, laminectomy, Epidural Fibrosis, EGR1.

## Abstract

**Rationale:** Epidural fibrosis is one of the contributors to failed back surgery syndrome (FBSS) with a high incidence of about 80,000 cases per year. The fibrosis spreads from the operative region to the dura mater or the nerve root and results in functional incapacity and pain after laminectomy. Our previous study showed that down-regulation of lncRNA-COX2 is involved in the epidural scar formation. However, it remains unknown whether lncRNA-COX2 participate in the fibroblast activation and epidural fibrogenesis.

**Methods:** LncRNA-COX2 and EGR1 expression were assessed by qRT-PCR and western blotting. Fibroblasts differentiation, proliferation and migration was determined by Collagen I/ɑ-SMA, 5-ethynyl-2'-deoxyuridine (EdU) and Transwell Assay respectively. Luciferase reporter assay was performed for the verification of target of LncRNA-COX2. Laminectomy was performed to establish the model of epidural fibrosis in mice. Epidural scar was evaluated by hematoxylin and eosin (HE) staining and Masson Trichrome staining.

**Results:** Based on the result of transcriptome profiling, we found LncRNA-COX2 was significantly decreased in epidural tissues after laminectomy and in activated fibrotic fibroblasts. *In vitro*, overexpression of LncRNA-COX2 suppressed epidural fibrogenesis by inhibiting fibroblasts differentiation, proliferation and migration. Mechanistically, LncRNA-COX2 functioned as competing endogenous RNA (ceRNA) of EGR1. Gain of LncRNA-COX2 significantly decreased the expression of EGR1 and showed anti-fibrotic effect while EGR1 was markedly increased after loss of LncRNA-COX2. *In vivo*, LncRNA-COX2 attenuated laminectomy-induced epidural fibrosis in mice.

**Conclusion:** In summary, the results demonstrated that LncRNA-COX2 showed anti-fibrotic effect by targeting EGR1 and identified LncRNA-COX2 as therapeutic molecule for preventing aberrant epidural fibrosis.

## Introduction

Failed back surgery syndrome (FBSS) refers to the residual symptoms and signs after lumbar laminectomy, discectomy or nerve root decompresssion, such as persistent pain or functional compromise in the waist, buttocks or lower limbs 1-3. Epidural fibrosis, cause of approximately 24% of FBSS with a high incidence per year 4, forms scar or tissue fibrosis within the scope of surgery, which surrounds and adheres to the epidural and/or lateral nerve roots 5,6, and results in functional incapacity and pain after spine surgery. Major progress has been made in understanding mechanisms of epidural fibrosis. A most relevant hypothesis is that the fibroblasts proliferated from their own mitosis and migrated from adjacent mesenchymal cells of perivertebral muscles and blood vessels to mechanically contract and compress the spinal roots and the neural sac 7,8. To control surgical injury repair under regenerative condition other than scar formation, approaches including various agents and materials have been demonstrated to attenuate the epidural fibrosis 9-13, but few of them have been applied into clinical practice due to uncertainty of its mechanism, thus highlighting the need for unremitting efforts to verify new biological pathways that can promise to be an adjunctive target for epidural fibrosis.

Long non-coding RNAs (LncRNAs) are a class of non-coding RNA that are more than 200-nucleotides in length without the ability to translate proteins 14-17. Increased evidence suggested that LncRNAs participated in the process of pathology and physiology by acting as a sponge of miRNAs or directly targeting genes 18-20. Recently, many studies demonstrated that LncRNAs are in association with fibrotic diseases 21-25. In our previous study, we screened differential LncRNAs and mRNAs via a microarray and identified that LncRNA-COX2 was significantly decreased whereas EGR1 showed remarkable higher expression after fibroblast differentiation 26. We showed that LncRNA-COX2 was highly expressed in the embryonic rat fibroblasts, but lowly expressed in the adult rat fibroblasts, which had negative correlations with the EGR1 level in embryonic and adult rat fibroblasts. In addition, the expression of EGR1 in the adult rat fibroblasts was remarkably higher than that in the embryonic rat fibroblasts after the activation with TGF-β. Meanwhile, the level of lncRNA-COX2 was lowered after the activation, especially in the adult rat fibroblasts. In-vivo results also demonstrated that the degree of fibroplasia was positively associated with EGR1 level and negatively correlated with lncRNA-COX2 level. In the present study, we further explored the potential relationship and mechanism between LncRNA-COX2 and EGR1 for epidural fibrosis.

## Methods

### Tissue specimen collection

We retrospectively collected cases of lumbar disc herniation or bulging among patients from Nanjing First Hospital. All patients provided informed consent, and the Ethic Committee of Nanjing Medical University approved the study according to the Declaration of Helsinki (No. 2017-467). Patients were eligible for inclusion in the study if they had undergone laminectomy to receive discectomy alone. Excluded were individuals who had received laminectomy plus fusion, who had local injection of drug at the operative site after laminectomy, who had substance abuse, who were predisposed to hypertrophic scarring and more extreme keloid formation in the skin incision site or who had malignant tumor.

### Primary Cell Extraction and Culture

Primary human epidural fibroblasts (HEFs) extracted from epidural tissue of patients who underwent revision surgery after laminectomy less than 7 days. The epidural scar tissue was soaked in 75% alcohol and separated into pieces and added with Trypsin-EDTA (ethylenediaminetetraacetic acid) digestive fluid (Keygen, Nanjing, China) at 37°C for 15 min. Then Fetal Bovine Serum was used to stop the digestion followed by centrifugation at a low speed (1300 rpm, 5 minutes) to obtain the cell suspension. The extracted cells were cultured in Dulbecco's Modifed Eagle's Medium (DMEM; KeyGen, Nanjing, China) solution containing 10% fetal bovine serum (FBS) and antibiotics. In all experiments, fibroblasts were used between passages 3 and 5.

### Animals and Grouping

Male C57BL/6J mice subjected to experiments, aged 8 weeks and weighed 20 g, were obtained from Experimental Animal Center of Nanjing Medical University. The mice were raised under normal environmental conditions (temperature 25 ± 1°C and humidity 60 ± 10%, 12 h / 12 h light and dark cycle per day) and were provided with adequate food and water. The mice were randomly divided into three groups: control group (tail intravenous injection of normal saline after laminectomy); LV-control group (treatment of control lentivirus by intravenous treatment after laminectomy); and LV-LncRNA-COX2 group (administration of lentivirus with LncRNA-COX2 by tail intravenous injection after laminectomy). Animal experiments were approved by the Institutional Animal Care and Use Committee (IACUC) of Nanjing Medical University, China (Approval No.: IACUC-1710004) and in conformity with the Guide for the Care and Use of Laboratory Animals (National Academies Press, 2011).

### Laminectomy and Drug Treatment

Preoperative preparation was as follows: Mice were anesthetized by intraperitoneal injection of pentobarbital sodium at a dose of 40 mg/kg and placed on the console at the prone position. The skin disinfected via iodophor following the dislodgement of back fur. The surgical procedure is summarized in the following: The incision of the back skin and the separation of the fascia and muscle on both sides of spinous process resulted in the exposure of intact lamina structure. Removal of the entire T10 lamina was from T9-T10 intervertebral space approach in succession. After hemostasis and saline flushing, suture of the tissue layer by layer and disinfection of skin were carried out. Cultured fibroblasts were activated via transforming growth factor-beta (TGF-β, 10 ng/ml, BioLegend, San Diego, CA, USA) for 24 h. The control group was treated with equivalent normal saline. To perform fibroblasts, the cells were firstly treated with DMEM without FBS for 6 hours. The plasmid of LncRNA-COX2, shRNA- LncRNA-COX2 or shRNA-EGR1 were separately mixed with Lipotectamine 2000 (Invitrogen, Carlsbad, CA, USA) and FBS-Free DMEM (Invitrogen, Carlsbad, CA, USA) for 20 min. And then the mixtures were added to different groups of fibroblasts for 8 hours. Finally, the fibroblasts obtained in the DMEM with FBS for 48 hours were employed for the subsequent experiments.

### Quantitative Polymerase Chain Reaction

TRIzol reagent (Invitrogen, Carlsbad, CA, USA) was added in the culture dish with 10% DMEM removed. The mixture was maintained for 10 minutes in room temperature then 200 μL chloroform was added and shaken fiercely for 30 s. Rested at room temperature for 5 minutes, the mixture was subjected to centrifugation (12000 rpm, 10 min) using a centrifugal machine. 500 μL liquid supernatant was transferred to RNase-free tube and added into 200 μL ethyl alcohol to be mixed together. The total mixture was transferred to a GenCleancolumn for 2 minutes and then centrifuged in 8000 rpm for 1 minute. With the supernatant abandoned, the sample was added into 600 μL buffer RWA and centrifuged (8000 rpm, 30 s). The above procedure was repeated, the supernatant was removed and the sample was centrifuged at 12000 rpm for 1 minute. 100 μL diethyl pyrocarbonate (DEPC)-H2O was added into the column and placed at 55°C -80°C for 2 minutes then the sample was centrifuged (12000 rpm, 1 min) again. Primer sequences were selected using Prime Script TM Master Mix (TaKaRa, Tokyo, Japan). qPCR was performed using a SYBR Green Mix (Vazyme Biotech, Nanjing, China). RNA sample was quantified and exhibited as the log-linear portion of the curve and was in contrast to an external calibration standard curve. The primers applied are as follows: LncRNA-COX2: Forward: 5'-AAGGAAGCTTGGCGTTGTGA-3, Reverse: 5'-GAGAGGTGAGGAGTCTTATG-3'; EGR1: Forward: 5'-ATTGGAGGAGATGATGCT-3', Reverse: 5'-AATTAGGAAATGTTGGTGC-3'; U6: Forward: 5'-CTCGCTTCGGCAGCACA-3', Reverse: 5'-AACGCTTCACGAAT-TTGCGT-3'.

### Luciferase Reporter Assay

Luciferase reporter assay was done by Dual Luciferase Reporter Gene Assay Kit (Beyotime, Shanghai, China) according to the manufacturer's protocol.

### Western blot

According to the previous study 27, protein from different groups of fibroblasts or epidural tissues were extracted using the Whole Cell Lysis Assay (Keygen, Nanjing, China). After the concentration was measured by bicinchoninic acid (BCA) method (Pierce, Rockford, IL, USA), Separation of proteins was done by 10% sodium dodecyl sulphate-polyacrylamide gel electrophoresis (SDS-PAGE) and proteins were transferred onto polyvinylidene fluoride (PVDF) membrane (Millipore, Billerica, MA, USA). 5% skim milk was used to seal the membrane and primary antibodies were employed to incubate the membrane at low temperature overnight. Finally, the PVDF membrane was washed by tris buffered saline-tween (TBST) for three times and incubate by secondary antibody for 1 hour at room temperature. The protein bans were visualized by electrochemiluminescence (ECL) and quantified by Image J.

### Immunofluorescence

The cultured HEFs of each group were re-suspended and inoculated on the slides in 12-well plates (WHB, Shanghai, China). After the cells grew to a satisfactory density, they were washed with PBS with culture medium eliminated. The HEFs treated with 4% paraformaldehyde (PFA) (Servicebio, Wuhan, China) for 20 min for immobilization. With PFA removed and washed by PBS (3×3 min), addition of 0.5% Triton X-100 (Biofroxx) brought about the permeation of the HEFs. The samples were sealed with the Serum (Beyotime, Shanghai, China) homologous to the secondary antibody for half an hour with Triton X-100 dumped and washed. Addition of the primary antibodies and incubation overnight at 4°C were subsequently done. After washing by phosphate buffered saline (PBS), the secondary antibodies at 1:500 (Alexa Fluor 488 Goat Anti-Rabbit IgG and catalog 136832, Jackson; Alexa Fluor 568 Donkey Anti-Rabbit IgG and catalog A10042, Invitrogen, Carlsbad, CA, USA) were added onto the HEFs respectively at 37°C for 30 min in the darkness. Then we removed the secondary antibody and washed samples with PBS. DAPI Fluoromount-G (Southernbiotech, Birmingham, AL, USA) was used to stain the cell nucleus. Finally, the samples were observed and the images captured under a fluorescence microscope.

The mice spines were fixed in the 4% PFA for 24 h. Then the samples were decalcified and dehydrated under ethanol with different concentration gradient, embedded in paraffin and cut into 3 μm sections via rotary microtome. After dewaxing treatment with xylene and different concentrations of ethanol, sections were immersed into citrate (PH 6.0) and boiled for 25 minutes. Then, the spine sections were blocked in BSA for an hour at room temperature, and incubated with primary antibodies (YAP/TAZ,1:500; fibronectin, 1:500; EGR1, 1:1000; collagen I, 1:500; α-SMA, 1:100). Fluorescent secondary antibodies at 1:500 (Goat-Anti-Rabbit IgG, catalog GB23303, Servicebio, Wuhan, China; Goat-Anti-Mouse IgG, catalog GB23301, Servicebio, Wuhan, China) were added onto the sections in dark for 50 minutes. CY3 reagent, FITC reagent and CY5 reagent (Servicebio, Wuhan, China) were added onto sections respectively and maintained for 10 minutes in dark room after eliminating primary antibodies. The sections were then washed by TBST and the DAPI Fluoromount-G was utilized to stain the nucleus. All the sections were observed and the images captured under a fluorescence microscope.

### Histological staining

After the spinal sections were fixed by 4% paraformaldehyde, hematoxylin and eosin (HE) staining and Masson Trichrome staining (Boster, Wuhan, China) were performed following deparaffinization with xylene and hydration according to the manufacturer's protocol.

### Transwell Assay

Trans-Well assay was conducted using 8 μm chambers (Corning, Corning, NY, USA). 100 μL HEFs (cell density 2×10^5^/well) with serum-free DMEM were transplanted into the upper chambers in a 24-well plate (WHB) following starvation treatment for 24 h and the lower chambers were respectively added in 500 μL of 10% DMEM with different treatment. After cells were cultured for 24 h, the upper chambers and complete medium in the lower chambers were removed. The unmigrated cells in upper layer were wiped with a cotton swab and 4% PFA was used to fix lower layer for 30 minutes. Then cells staining were conducted using 0.1% crystal violet for 30 minutes. Digital images were collected under a microscope.

### 5-ethynyl-2'-deoxyuridine (EdU) Assay

Fibroblasts were cultured in 24-well plates with satisfied density. Proliferation was determined by Cell-Light EdU kit (Keygen, Nanjing, China). In brief, culture solution containing EdU reagent was used to incubate fibroblasts and DAPI staining for nucleus was performed after cells were fixed.

### Statistical analysis

Statistical Product and Service Solutions (SPSS) 19.0 software (IBM, Armonk, NY, USA) was employed for the Statistical analysis. All quantitative data were expressed as mean ± standard deviation of three independent experiments. Two-tailed t-test and one-way analysis of variance (ANOVA) followed by Bonferroni's correction (post hoc test) were respectively conducted for the comparisons in two and multiple groups. *P*-values < 0.05 were considered statistically significant.

## Results

### LncRNA-COX2 inhibits TGF-β-induced fibroblast differentiation, proliferation and migration

**Figure 1:**
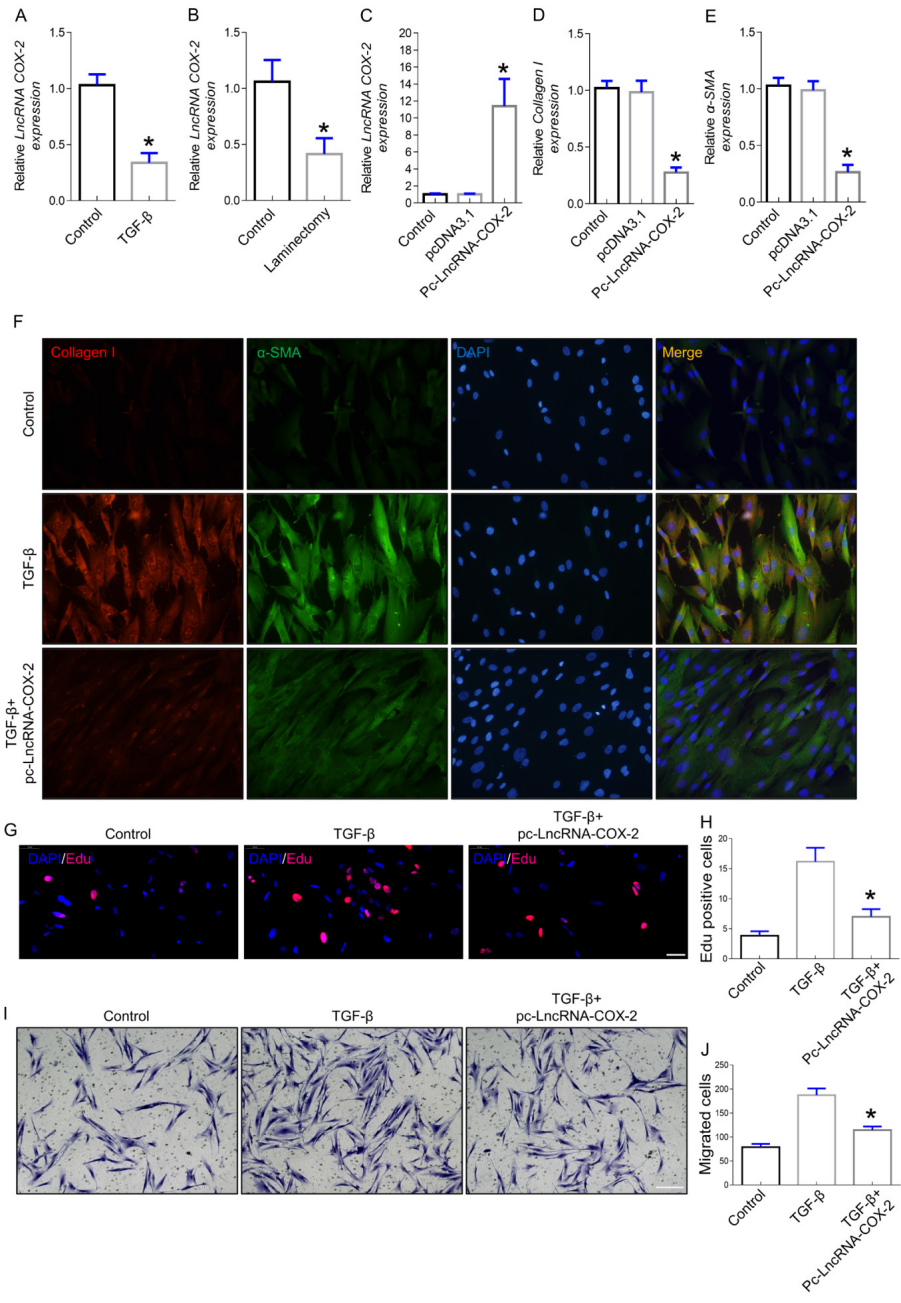
** LncRNA-COX2 inhibits fibrogenesis in epidural fibroblasts.** (**A**) Basal LncRNA-COX2 expression in fibroblasts treated with TGF-β or not and (**B**) epidural tissues from surgical site and normal region of mice. qRT-PCR analysis of the expression of LncRNA-COX2 (**C**), Collagen I (**D**), α-SMA (**E**) in fibroblasts after transfection of LncRNA-COX2. (**F**) Immunofluorescence analysis of collagen I and α-SMA expression in TGF-β-cultured fibroblasts treated with LncRNA-COX2. (**G**) EdU analysis showing proliferative fibroblasts after TGF-β or LncRNA-COX2 treatment. Scale bar = 50 μm. (**H**) Quantitative analysis of proliferative fibroblasts in indicated groups. (**I**) Transwell assay showing migrated fibroblasts after TGF-β or LncRNA-COX2 treatment. Scale bar = 100 μm. (**J**) Quantitative analysis of migrated fibroblasts in indicated groups. *P<0.05.

Increased evidence indicated that lncRNAs played crucial roles in the occurrence and development of fibrotic diseases. In our previous study, we found that LncRNA-COX2 was significantly upregulated in differentiated fibroblasts 26. To further ascertain whether LncRNA-COX2 participates in induction of epidural fibroblasts to become activated myofibroblasts and secretion of extracellular matrix (ECM), we investigated the expression of fibrogenesis-associated genes. Myofibroblast, characterized by expression of ɑ-SMA, produces ECM containing collagen I that is fundamental for ECM deposition 28,29. Here, we found LncRNA-COX2 was significantly decreased in fibroblasts treated with TGF-β (Figure 1A) and in epidural tissues underwent laminectomy (Figure 1B). After overexpression of LncRNA-COX2 (Figure 1C), Collagen I and ɑ-SMA were remarkably reduced (Figure 1D-F). The well-known mechanisms of epidural fibrogenesis involve fibroblasts proliferation in resident location and migration from peripheral zone of operation 30-32. In the present study, we observed the effect of LncRNA-COX2 on epidural fibroblast proliferation. The EdU assay indicated that LncRNA-COX2 could markedly decreased the number of proliferative fibroblasts induced by TGF-β (Figure 1G, 1H). Besides, Transwell chamber assay was employed to detect the chemotaxis migration of fibroblasts. We found that fibroblasts treated with TGF-β revealed greater migration than control, which was blocked by LncRNA-COX2 (Figure 1I, 1J).

### EGR1 is a direct target of LncRNA-COX2

**Figure 2:**
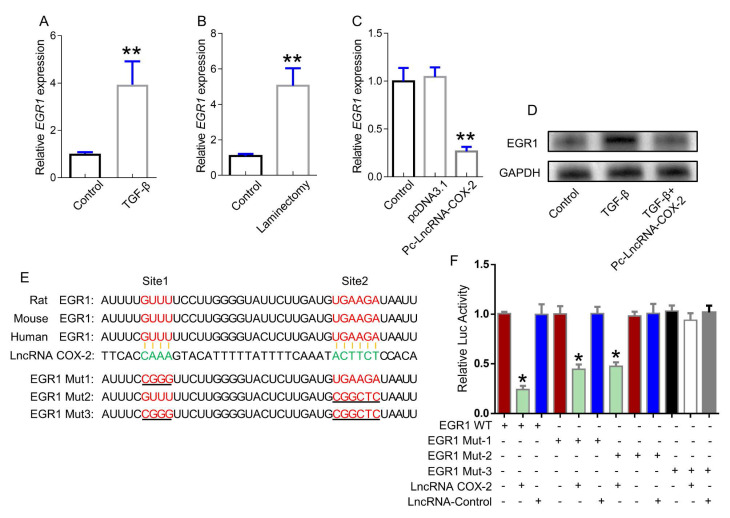
** LncRNA-COX2 interacts with EGR1.** (**A**) qRT-PCR analysis of the expression of EGR1 in fibroblasts treated with TGF-β or not and (**B**) epidural tissues from surgical site and normal region of mice. (**C**) qRT-PCR analysis of EGR1 level in fibroblasts after LncRNA-COX2 overexpression. (**D**) Western blot analysis of EGR1 in fibroblasts in treated with TGF-β or after LncRNA-COX2 overexpression. (**E**) Predicted binding sites of LncRNA-COX2 and EGR1. Mut-1, mutated site 1; Mut-2, mutated site 2; Mut-3, mutated both sites 1 and 2. *P<0.05, **P<0.01.

EGR1 was demonstrated to be a key role in epidural fibrosis. In our study, EGR1 was significantly increased in activated fibroblasts and injured epidural tissues (Figure 2A, 2B), which was inhibited by LncRNA-COX2 treatment (Figure 2C, 2D). To further clarify the potential mechanism regulating fibrogenesis of LncRNA-COX2, we explored the underlying relationship between LncRNA-COX2 and EGR1. As shown in Figure 2E, the EGR1 contained a complementary base sequence for LncRNA-COX2, which was broadly conserved among rat, mouse and human. The results indicated that LncRNA-COX2 could inhibit the luciferase activity with EGR1-WT and mutated EGR1 with one mutation in binding sites in fibroblasts. However, LncRNA-COX2 had no significant effect on two-mutated sites of EGR1 (Figure 2F).

### LncRNA-COX2 inhibition promotes fibrogenesis by regulating EGR1

**Figure 3:**
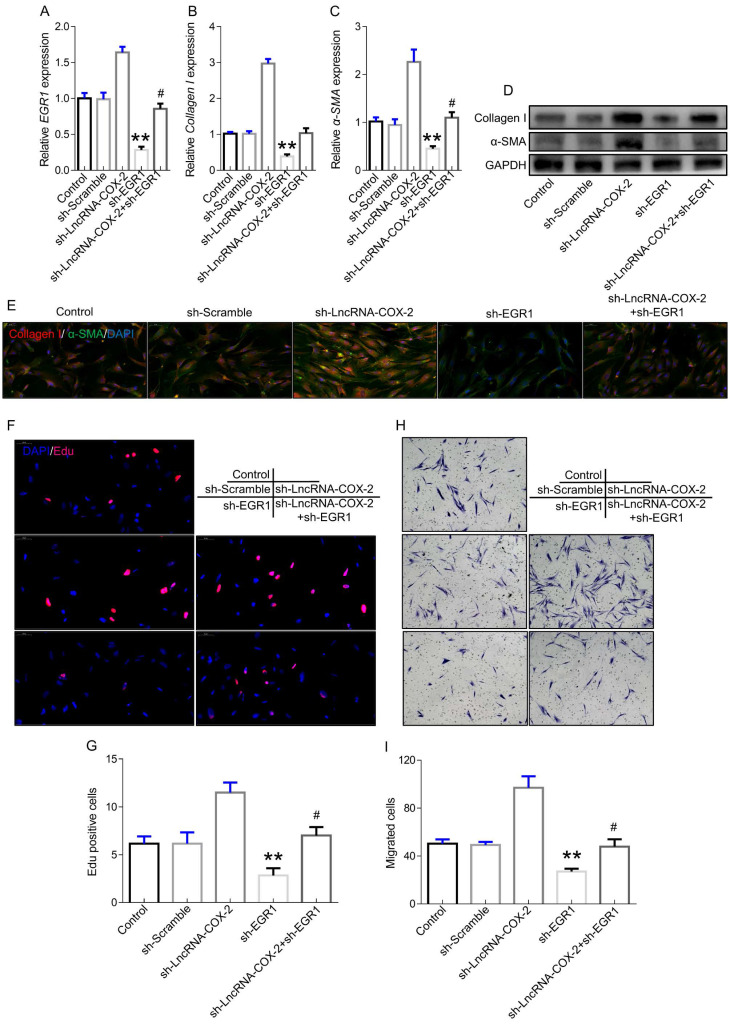
** Silencing LncRNA-COX2 promotes fibroblasts activation after EGR1 is suppressed.** qRT-PCR analysis of EGR1 (**A**), Collagen I (**B**), α-SMA (**C**) in fibroblasts after EGR1 or LncRNA-COX2 inhibition. (**D**) Western blot analysis of EGR1, Collagen I, α-SMA in fibroblasts after EGR1 or LncRNA-COX2 inhibition. (**E**) Immunofluorescence analysis of collagen I and α-SMA expression in cultured fibroblasts knockdown of EGR1 or LncRNA-COX2. EdU assay (**F**) and Transwell assay (**H**) of fibroblasts in indicated groups. (**G**) Quantitative analysis of proliferative cells of (**F**). (**I**) Quantitative analysis of migrated cells of (**H**). ^#^*P*<0.05, **P<0.01. Difference was analyzed by one-way ANOVA.

To further confirm the role of LncRNA-COX2-EGR1 axis in fibrogenesis, we performed the subsequent experiments. EGR1 was significantly increased in fibroblasts after LncRNA-COX2 was inhibited and the inhibitory effect of sh-EGR1 was counteract by suppression of LncRNA-COX2 (Figure 3A). Additionally, Collagen I and ɑ-SMA showed lower expression in sh-EGR1 group compared to control group. However, after inhibition of LncRNA-COX2, fibrogenesis was remarkably promoted (Figure 3B-3E). The results of EdU analysis showed elevated proliferative levels after LncRNA-COX2 was inhibited while knockdown of EGR1 significantly decreased EdU-positive cell number and inhibited proliferative effect after suppression of LncRNA-COX2 (Figure 3F, 3G). Transwell chamber assays were performed to determine the effect of LncRNA-COX2 and EGR1 on directed migration of fibroblast. We found that after LncRNA-COX2 was down-regulated fibroblasts showed more migrated cells than control group, and migration was blocked following EGR1 inhibition (Figure 3H, 3I).

### Administration of LncRNA-COX2 decreases degrees of epidural fibrosis

**Figure 4:**
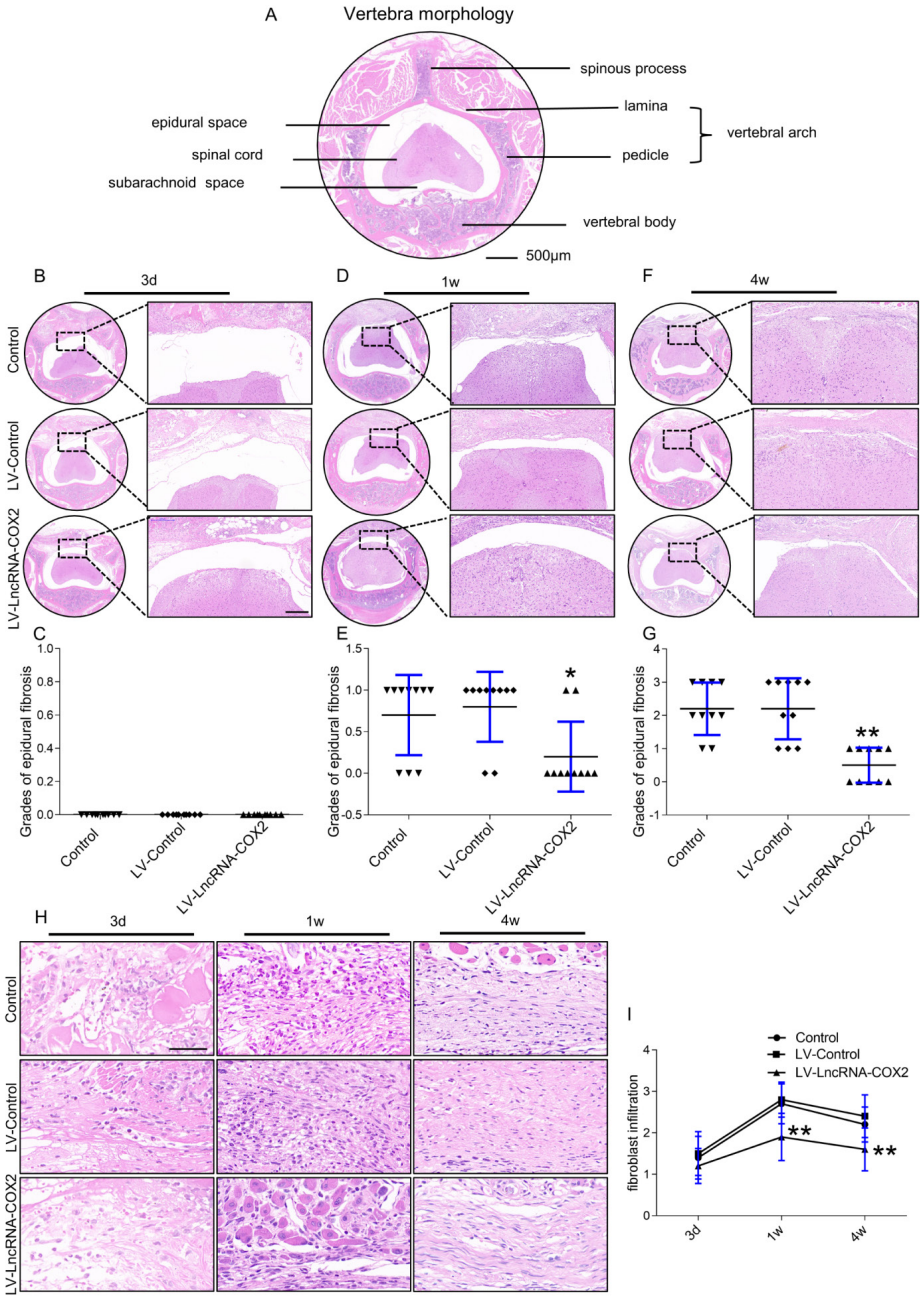
** Degree of epidural fibrosis is markedly decreased after LncRNA-COX2 treatment.** (**A**) Axial image of thoracic vertebrae in mice. Scale bar: 500μm. HE staining of representative images of the vertebrae morphology showing epidural wound injury site after laminectomy in different groups at the time of 3 days (**B**), 1 week (**D**), 4 weeks (**F**). Scale bar: 200 μm. Grades of epidural fibrosis in different groups at the time of 3 days (**C**), 1 week (**E**), 4 weeks (**G**) (n = 10). *P<0.05. **P<0.01. (H) Histological assessment of fibroblast infiltration at 3 days, 1 week and four weeks post-operation by HE staining analysis. Scale bar: 50 μm. (**I**) Fibroblast infiltration grades in epidural tissues in control group, LV-control group and LV- LncRNA-COX2 group at three different times (n = 10). *P<0.05, **P<0.01. Difference was analyzed by one-way ANOVA.

Since our *in vitro* findings implicated anti-fibrosis role of LncRNA-COX2 in fibrotic fibroblasts, we next decided to explore its characters in fibrogenesis *in vivo*. Epidural fibrosis is mainly caused by laminectomy generally applied for spine disorder, including spinal stenosis and herniated disks. Laminectomy, a procedure that removes lamina to widen the spinal canal and create more space for the spinal nerves and thecal sac, may lead to residual scar tissue formation and subsequent post-laminectomy syndrome, and thus the epidural fibrosis model induced by laminectomy is well widespread and recognized. Histological examination was done to investigate the diverse degrees of epidural fibrosis. As shown in Figure 4A, the vertebra of mouse consists of the anterior vertebral body and the posterior arch that form a conical foramen to accommodate the spinal cord. In laminectomy surgery, we removed spinous process and lamina. Then we observed adhesion tissues extending to the dura mater at different time points among the control, LV-control and LV- LncRNA-COX2 groups. Epidural adhesion tissues were gradually expanded to the dura mater of the surgical region in each group as time went by. For staging according to fibrosis score 6 three days after surgery, grades of epidural fibrosis were 0 with only loose and little fibrosis tissue formed above the spinal cord in all subjects (Figure 4B and 4C). However, epidural fibrosis tissues began to adhere to the spinal cord in the control and LV-control groups and there was a significant lower average grade in the LV- LncRNA-COX2 group a week post-the surgery (Figure 4D and 4E). At four weeks, the epidural scar already spread to the dura mater and tended to compress the spinal cord in the control and LV-control groups; on the contrary, the LV- LncRNA-COX2 group showed space between the dura mater with no adhesion to the spinal cord. Besides, remarkable differences were also noted between the LV-control group and the LV- LncRNA-COX2 group (Figure 4F and 4G). Fibroblast density is used as an index to evaluate epidural fibrosis 6. Extent of fibrosis is positively correlated with density of fibroblast. All the three groups displayed low fibroblast density with no significant difference in grades of fibroblast infiltration in epidural fibrosis tissues three days after laminectomy. The density significantly increased after one week, at which enormous amounts of fibroblasts proliferated from resident sites and recruited from perivertebral muscles and blood to participate in injured tissue repair (Figure 4H). Use of LncRNA-COX2 was sufficient to significantly decrease density at the end point of one week. In the final formative stages of fibrosis, grade of fibroblast infiltration in the LV- LncRNA-COX2 group was found to be markedly reduced as opposed to that in the control and LV-control groups. Additionally, there was no remarkable difference of fibroblast density either with or without LV-control (Figure 4I). These results suggest that LncRNA-COX2 ameliorates the epidural fibrosis reflected in the decreased fibrosis grade and fibroblast density.

### LncRNA-COX2 suppresses epidural fibrogenesis initiation

**Figure 5:**
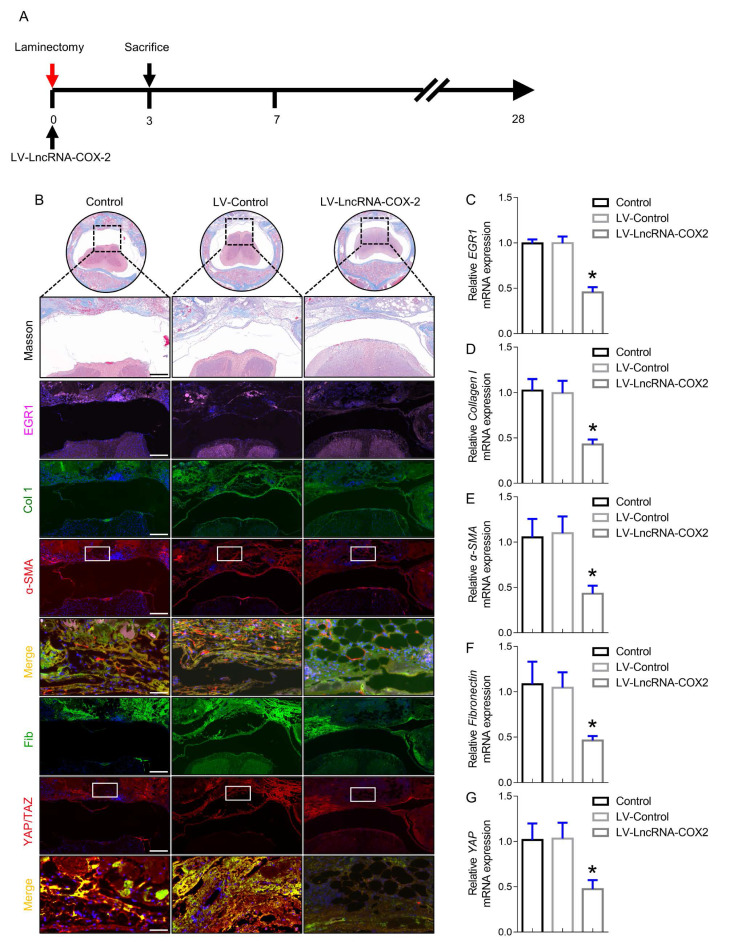
** LncRNA-COX2 inhibits epidural fibrogenesis initiation.** (**A**) Schematic illustrating the timelines for *in vivo* introduction of LncRNA-COX2 and determination of experimental sacrifice time in the laminectomy model of epidural fibrosis. (**B**) Cross sections of the vertebral column stained with Masson's trichrome staining to show collagen deposition (blue) and stained with the fibrotic indications. (n = 3 mice per group) at day 3. Scale bars: 200 μm. Higher-power image scale bars: 50 μm. (**C**) EGR1 level of epidural tissues in mice treated with saline, LV-control or LV-LncRNA-COX2. Effect of LncRNA-COX2 on the epidural fibrogenesis initiation reflected by the mRNA expression of fibrotic markers (Collagen I, α-SMA, YAP and Fibronectin) (**D**-**G**). In C-G, each symbol represents an individual mouse. *P<0.05. Difference was analyzed by one-way ANOVA.

To analyze the mechanism of LncRNA-COX2 responsible for the occurrence and development of epidural fibrosis *in vivo*, we sought to determine whether genetic inhibition of EGR1 with LncRNA-COX2 was able to ameliorate laminectomy-induced early fibrotic phase at 3 days after surgery. As shown in Figure 5A, LncRNA-COX2 was administered after laminectomy. Although surgical injury was terminated, epidural fibrosis initiation still did not cease or regress and had self-sustaining ability. In this inflammatory stage, morphological analysis showed that red blood cells induced by injury were distributed in the surgical region with mild fibrogenesis determined by Trichrome staining (Figure 5B). EGR1 was continuously expressed on the 3rd day in control and LV-control groups. In contrast, we found that epidural tissues of mice treated with LncRNA-COX2 displayed decreased EGR1 content (Figure 5B, 5C). Importantly, immunofluorescence analysis suggested that more ɑ-SMA, collagen I, fibronectin and YAP/TAZ positive cells were distributed in the fibrotic regions of epidural tissues in the control and LV-control groups than in the LV- LncRNA-COX2 group. Consistent with the results of *in vivo* experiments, epidural tissues from the LncRNA-COX2 treated mice demonstrated markedly decreased expression of mRNA encoding the fibrosis-associated genes (Figure 5D-5G).

### Progression of epidural fibrosis is inhibited after LncRNA-COX2 introduction

**Figure 6:**
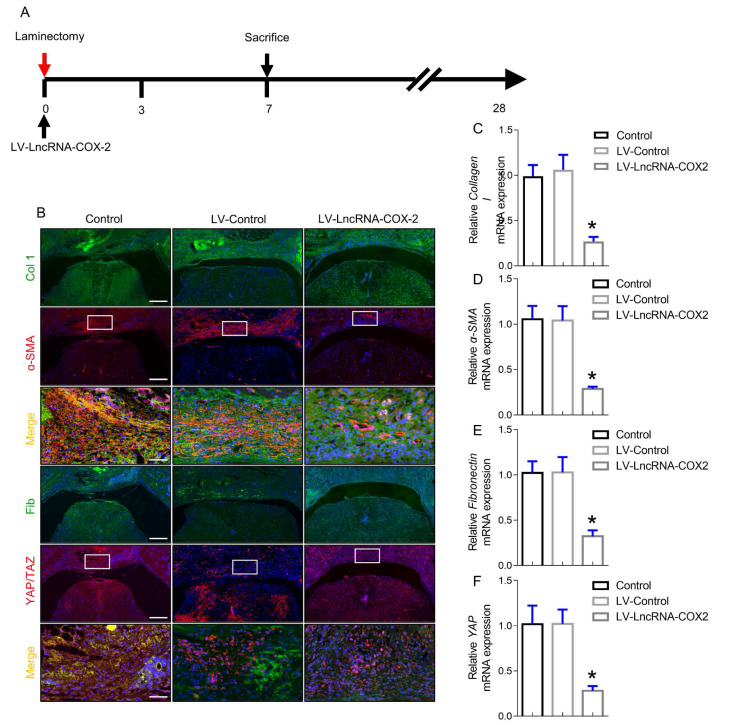
**LncRNA-COX2 weakens the progression of epidural fibrosis.** (**A**) Schematic illustrating the timelines for *in vivo* use of LncRNA-COX2 and decision of experimental end points in the laminectomy model of epidural fibrosis. (**B**) Cross sections of the vertebral column stained with Masson's trichrome staining to show collagen deposition (blue) and stained with the fibrotic indications. (n = 3 mice per group) at day 7. Scale bars: 200 μm. Higher-power image scale bars: 50 μm. Effect of LncRNA-COX2 on the epidural fibrogenesis initiation reflected by the mRNA expression of fibrotic markers Collagen I (**C**), α-SMA (**D**), YAP (**E**) and Fibronectin (**F**). In C-F, each symbol represents an individual mouse. *P<0.05. Difference was analyzed by one-way ANOVA.

The intermediate stage of wound repair, defined as new tissue formation phase, involves proliferation and migration of fibroblasts, ECM deposition, and angiogenesis. It usually happens a week following injury characterized by cell-cell, cell-matrix and epidermal-mesenchymal interactions 33. After laminectomy, fibroblasts from the epidural space of the wound or from the blood vessel differentiate into myofibroblasts. In the wound injury site, fibroblasts and myofibroblasts interact and generate collagen composed ECM, which is a loopback driver of progressive fibrosis in a YAP dependent manner 34,35. Since YAP is a key role of cell-cell and cell-matrix interactions in regulating fibrogenesis, we examined the content of YAP of epidural fibrosis 7 days post laminectomy. Coinciding with *in vitro* results, YAP expression was markedly decreased after LncRNA-COX2 treatment. Notably, ɑ-SMA, collagen I and fibronectin expressing cells in the epidural tissues was significantly decreased in the LV- LncRNA-COX2 group (Figure 6B). Furthermore, LncRNA-COX2 markedly reduced expression of the fibrosis-associated genes mRNA compared to the control and LV-control groups (Figure 6C-6F).

### *In vivo* treatment with LncRNA-COX2 protects mice from laminectomy-induced epidural fibrosis

**Figure 7:**
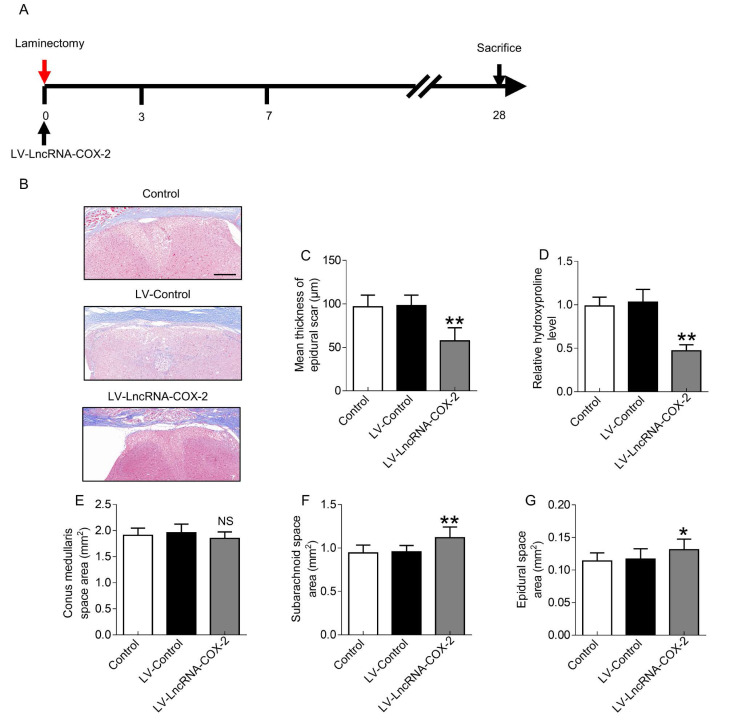
** LncRNA-COX2 decreases laminectomy-induced epidural scar formation.** (**A**) Schematic illustrating the timelines for *in vivo* administration of LncRNA-COX2 and determination of experimental end points, and for the final phase in the laminectomy model of epidural fibrosis. (**B**) Cross sections of the vertebral column stained with Masson's trichrome staining to show collagen deposition (blue) (n = 3 mice per group) at day 28. Scale bar: 200 μm. The effect of epidural scar on spinal cord after oral administration of saline, LV-control or LV- LncRNA-COX2 reflected by (**C**) mean thickness of epidural scar, (**D**) relative hydroxyproline content, (**E**) conus medullaris space area, (**F**) subarachnoid space area and (**G**) epidural space area. *P<0.05. **P<0.01. NS means not significant. Difference was analyzed by one-way ANOVA.

Since LncRNA-COX2 was able to inhibit epidural fibrogenesis initiation and progression, we sought to investigate whether treatment with LncRNA-COX2 could ameliorate epidural scar established as the provisional ECM degraded and remodeled in the final fibrotic maturation phase at 28 days after surgery (Figure 7A). As expected, laminectomy resulted in massive epidural fibrosis tissue on the spinal cord with mean thickness of epidural scar increased. Conditional introduction of LncRNA-COX2 caused a significant decrease of collagenous matrix deposition as evidenced by attenuated Masson's trichrome staining, reduced scar thickness and decreased epidural hydroxyproline content as opposed to the control and LV-control groups (Figure 7B-7D). In addition, conus medullaris, subarachnoid, and epidural space areas were used to evaluate the compression effect as previously described 12. As shown in Figure 7E, difference in the areas of the conus medullaris space among the control, the LV-control and the LV- LncRNA-COX2 groups were not significant. However, treatment with LncRNA-COX2 to the mice undergoing laminectomy surgery resulted in a significant increment in the area of subarachnoid space (Figure 7F) and epidural space (Figure 7G). These results demonstrate that invasive fibrosis in the presence or absence of LncRNA-COX2 is insufficient to impact the area of conus medullaris space, whereas inhibition of EGR1 by LncRNA-COX2 is able to ameliorate the compression effect of epidural scar induced by laminectomy.

## Discussion

Fibrosis is a progressive disease with excessive deposition of extracellular matrix (ECM) composed of fibrillar collagens, EDTA-fibronectin and SMA-stress fiber 27,36-39. Sustained fibroblast proliferation, activation and migration, generally identified as wound healing response, continually happen after tissue injury 40,41. Healing by epidural fibrosis instead of regeneration may place burden on spinal cord and nerve root after spinal surgery 42.

Few studies have evaluated the relationship of LncRNAs in epidural fibrosis. In the previous study, we found that low expression of LncRNA-COX2 and high expression of EGR1 was associated with a significantly higher epidural fibrosis grade 26. There have been conflict and controversy about the role of LncRNAs in varieties of fibrogenesis. For example, some lncRNAs have been clarified to show anti-fibrosis effect in renal fibrosis 43, pulmonary fibrosis 44, skin fibrosis 45. On the contrary, some lncRNAs are demonstrated to have pro-fibrosis characteristics in cardiac fibrosis 46, liver fibrosis 47, skeletal muscle fibrosis 48.

**Figure 8:**
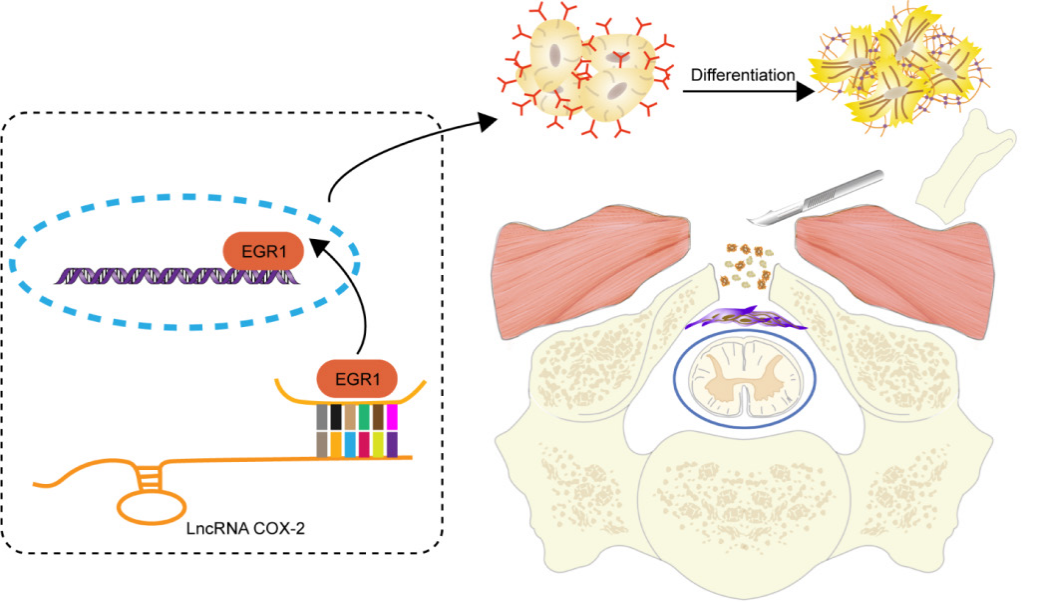
Schematic diagram illustrates the role of LncRNA-COX2 in the progression of laminectomy-induced epidural scar formation.

In the present study, we further validated that LncRNA-COX2 baseline level was remarkably lower in the surgical site as opposed to normal region of mice undergoing laminectomy. It is important for fibroblasts to differentiate into myofibroblasts, which produces collagen deposition to repair the injury site. In parallel experiments *in vitro*, LncRNA-COX2 fell when fibroblasts were activated and fully mature into myofibroblasts. Genetically promotion of LncRNA-COX2 level simultaneously promoted myofibroblast dedifferentiation and inhibited ECM formation, as reflected by down-regulated ɑ-SMA and collagen I. Our results linking upregulation of LncRNA-COX2 to myofibroblast dedifferentiation and the mechanism by which LncRNA-COX2 inhibits the differentiation remain to be examined. Besides, Fibroblasts undergo remarkable alteration in phenotype and gene expression resulting in cell proliferation and migration. We found LncRNA-COX2 could suppress fibroblast proliferation and migration induced by TGF-β. Growing evidence indicated that EGR1 was involved in organ fibrosis 49 and we further confirmed fibrosis was inhibited after EGR1 was decreased. Our study showed that LncRNA-COX2 could act as a sponge of EGR1 and overexpression of LncRNA-COX2 significantly inhibited fibroblast differentiation and decreased ECM formation by suppressing EGR1 expression while silencing LncRNA-COX2 increased EGR1 level. To our knowledge, it is the first study to demonstrate the cellular mechanism of LncRNA for epidural fibrosis in the injury tissues of mice by laminectomy-induced model. Reportedly, the response to wound injury generally falls into three overlapping but diverse stages: inflammation, new tissue formation, and remodeling 50,51. According to the concept, specimens were harvested on days 3, 7 and 28 to examine the effect of LncRNA-COX2 on three different periods of epidural tissue injury. Our present results showed that during inflammation phase, hemorrhage in the epidural injury site just occurred beside the dura mater. We demonstrated a dramatic increase of fibroblasts in laminectomy-induced mice in both control and LV-control groups. LncRNA-COX2 significantly inhibited the fibroblasts activation and migration. Furthermore, LncRNA-COX2 inhibited laminectomy-induced EGR1 expression, which resulted in the dedifferentiation of myofibroblast reflected by decreased ɑ-SMA level and reduced ECM position indicated by lessened collagen, YAP and fibronectin. New tissue formation stage, characterized by various types of cells proliferation and migration, frequently happens about 2-10 days after injury. In consistence with this, fibroblasts which migrated from the surrounding environment differentiated into myofibroblasts and produced ECM composed mainly of collagen 52. Introduction of LncRNA-COX2 for 7 days still exhibited antifibrotic effect by reducing the fibrosis-associated genes and inhibiting the migration of fibroblasts. Remodeling stage occurs about 2-3 weeks after injury. When wound injury repair ensues after a single discrete injury, ECM remodeling eventually leads to the formation of mature scar composed of highly cross-linked collagen and other ECM proteins. As the remodeling proceeds, the amounts of myofibroblasts significantly decrease just as observed on day 28 after laminectomy. In the present study, we found that the compression effect was exacerbated in the LV-control group compared to that in the celecoxib group whereas no remarkable difference was observed between the control group and the LV-control group. Our results manifest that administering LncRNA-COX2 exerts inhibitory effect on epidural fibrosis and genetic inhibition of EGR1 was sufficient to provide remarkable protection in all the fibrotic end points detected (Figure 8). However, imitations still existed in our present study. As we know, it's better to further verify the effects of LncRNA-COX2 and EGR1 on epidural fibrosis and to explore the deeper molecular mechanism in EGR1-knockout animal models. In our future study, we will try to establish the EGR1-knockout animal models for the further verification.

## Conclusions

In conclusion, the present study characterized LncRNA-COX2-EGR1 axis as a key role of fibroblast differentiation, proliferation and migration and provided potential mechanism for epidural fibrosis. Our *in vivo* and invitro results indicated that inhibition of EGR1 by LncRNA-COX2 may be novel therapeutic strategy of epidural fibrosis.

## Abbreviations

FBSS: Failed back surgery syndrome
LncRNAs: Long non-coding RNAs
HEFs: human epidural fibroblasts
EDTA: ethylenediaminetetraacetic acid
DMEM: Dulbecco's Modifed Eagle's Medium
FBS: fetal bovine serum
IACUC: Institutional Animal Care and Use Committee
TGF-β: transforming growth factor-beta
DEPC: diethyl pyrocarbonate
BCA: bicinchoninic acid
SDS-PAGE: sodium dodecyl sulphate-polyacrylamide gel electrophoresis
PVDF: polyvinylidene fluoride
TBST: tris buffered saline-tween
ECL: electrochemiluminescence
PFA: paraformaldehyde
PBS: phosphate buffered saline
HE: hematoxylin and eosin
EdU: 5-ethynyl-2'-deoxyuridine
SPSS: Statistical Product and Service Solutions
ANOVA: analysis of variance
ECM: extracellular matrix

## References

[1] Fritsch EW, Heisel J, Rupp S (1996). The failed back surgery syndrome: reasons, intraoperative findings, and long-term results: a report of 182 operative treatments. Spine (Phila Pa 1976).

[2] Talbot L (2003). "Failed back surgery syndrome". BMJ.

[3] Amirdelfan K, Webster L, Poree L, Sukul V, McRoberts P (2017). Treatment Options for Failed Back Surgery Syndrome Patients With Refractory Chronic Pain: An Evidence Based Approach. Spine (Phila Pa 1976).

[4] Samy AM, Hardy RJ (1999). Epidural fibrosis and the failed back surgery syndrome: history and physical findings. Neurol Res.

[5] Zhang C, Kong X, Ning G, Liang Z, Qu T, Chen F, Cao D, Wang T, Sharma HS, Feng S (2014). All-trans retinoic acid prevents epidural fibrosis through NF-kappaB signaling pathway in post-laminectomy rats. Neuropharmacology.

[6] Tanriverdi O, Yilmaz I, Adilay HU, Gunaldi O, Erdogan U, Gungor A, Kilic M, Tanik C (2019). Effect of Cetuximab on the Development of Epidural Fibrosis Based on CD105 and Osteopontin Immunohistochemical Staining. Spine (Phila Pa 1976).

[7] Kurt G, Aytar MH, Dogulu F, Cemil B, Erdem O, Baykaner MK, Ceviker N (2008). A comparison of the local effectiveness of mitomycin C, aprotinin, and Adcon-L in experimental peridural fibrosis. Surg Neurol.

[8] Bora H, Aykol SV, Akyurek N, Akmansu M, Ataoglu O (2001). Inhibition of epidural scar tissue formation after spinal surgery: external irradiation vs. spinal membrane application. Int J Radiat Oncol Biol Phys.

[9] Cunningham BW, Seiber B, Riggleman JR, Van Horn MR, Bhat A (2019). An investigational study of a dual-layer, chorion-free amnion patch as a protective barrier following lumbar laminectomy in a sheep model. J Tissue Eng Regen Med.

[10] Liu J, Ni B, Zhu L, Yang J, Cao X, Zhou W (2010). Mitomycin C-polyethylene glycol controlled-release film inhibits collagen secretion and induces apoptosis of fibroblasts in the early wound of a postlaminectomy rat model. Spine J.

[11] Su C, Sui T, Zhang X, Zhang H, Cao X (2012). Effect of topical application of mitomycin-C on wound healing in a postlaminectomy rat model: an experimental study. Eur J Pharmacol.

[12] Wu CY, Huang YH, Lee JS, Tai TW, Wu PT, Jou IM (2016). Efficacy of topical cross-linked hyaluronic acid hydrogel in preventing post laminectomy/laminotomy fibrosis in a rat model. J Orthop Res.

[13] Shi K, Wang F, Xia J, Zuo B, Wang Z, Cao X (2019). Pirfenidone inhibits epidural scar fibroblast proliferation and differentiation by regulating TGF-beta1-induced Smad-dependent and -independent pathways. Am J Transl Res.

[14] Mercer TR, Dinger ME, Mattick JS (2009). Long non-coding RNAs: insights into functions. Nat Rev Genet.

[15] Ponting CP, Oliver PL, Reik W (2009). Evolution and functions of long noncoding RNAs. Cell.

[16] Wang KC, Chang HY (2011). Molecular mechanisms of long noncoding RNAs. Mol Cell.

[17] Batista PJ, Chang HY (2013). Long noncoding RNAs: cellular address codes in development and disease. Cell.

[18] Chen Y, Li S, Zhang Y, Wang M, Li X, Liu S, Xu D, Bao Y, Jia P, Wu N, Lu Y, Jia D (2021). The lncRNA Malat1 regulates microvascular function after myocardial infarction in mice via miR-26b-5p/Mfn1 axis-mediated mitochondrial dynamics. Redox Biol.

[19] Cheng X, Shihabudeen HAM, Moran M, Viana MP, Schlichte SL, Zimmerman MC, Khalimonchuk O, Feinberg MW, Sun X (2021). Long non-coding RNA Meg3 deficiency impairs glucose homeostasis and insulin signaling by inducing cellular senescence of hepatic endothelium in obesity. Redox Biol.

[20] Su SC, Yeh CM, Lin CW, Hsieh YH, Chuang CY, Tang CH, Lee YC, Yang SF A novel melatonin-regulated lncRNA suppresses TPA-induced oral cancer cell motility through replenishing PRUNE2 expression. J Pineal Res 2021: e12760.

[21] Sun J, Jin T, Su W, Guo Y, Niu Z, Guo J, Li L, Wang J, Ma L, Yu T, Li X, Zhou Y, Shan H, Liang H The long non-coding RNA PFI protects against pulmonary fibrosis by interacting with splicing regulator SRSF1. Cell Death Differ 2021.

[22] Luo S, Zhang M, Wu H, Ding X, Li D, Dong X, Hu X, Su S, Shang W, Wu J, Xiao H, Yang W, Zhang Q, Zhang J, Lu Y, Pan Z (2021). SAIL: a new conserved anti-fibrotic lncRNA in the heart. Basic Res Cardiol.

[23] Pachera E, Assassi S, Salazar GA, Stellato M, Renoux F, Wunderlin A, Blyszczuk P, Lafyatis R, Kurreeman F, de Vries-Bouwstra J, Messemaker T, Feghali-Bostwick CA, Rogler G, van Haaften WT, Dijkstra G, Oakley F, Calcagni M, Schniering J, Maurer B, Distler JH, Kania G, Frank-Bertoncelj M, Distler O (2020). Long noncoding RNA H19X is a key mediator of TGF-beta-driven fibrosis. J Clin Invest.

[24] Wasson CW, Abignano G, Hermes H, Malaab M, Ross RL, Jimenez SA, Chang HY, Feghali-Bostwick CA, Del GF (2020). Long non-coding RNA HOTAIR drives EZH2-dependent myofibroblast activation in systemic sclerosis through miRNA 34a-dependent activation of NOTCH. Ann Rheum Dis.

[25] Xiao Y, Liu R, Li X, Gurley EC, Hylemon PB, Lu Y, Zhou H, Cai W (2019). Long Noncoding RNA H19 Contributes to Cholangiocyte Proliferation and Cholestatic Liver Fibrosis in Biliary Atresia. Hepatology.

[26] Qian ZY, Jiang F, Tang J, Ge DW, Chen HT, Zheng SN, Cao XJ, Ge YB, Yang L (2019). Potential roles of lncRNA-Cox2 and EGR1 in regulating epidural fibrosis following laminectomy. Eur Rev Med Pharmacol Sci.

[27] Qian Z, Chang J, Jiang F, Ge D, Yang L, Li Y, Chen H, Cao X (2020). Excess administration of miR-340-5p ameliorates spinal cord injury-induced neuroinflammation and apoptosis by modulating the P38-MAPK signaling pathway. Brain Behav Immun.

[28] Chen H, Qian Z, Zhang S, Tang J, Fang L, Jiang F, Ge D, Chang J, Cao J, Yang L, Cao X (2021). Silencing COX2 blocks PDK1/TRAF4-induced AKT activation to inhibit fibrogenesis during skeletal muscle atrophy. Redox Biol.

[29] Liu F, Lagares D, Choi KM, Stopfer L, Marinkovic A, Vrbanac V, Probst CK, Hiemer SE, Sisson TH, Horowitz JC, Rosas IO, Fredenburgh LE, Feghali-Bostwick C, Varelas X, Tager AM, Tschumperlin DJ (2015). Mechanosignaling through YAP and TAZ drives fibroblast activation and fibrosis. Am J Physiol Lung Cell Mol Physiol.

[30] Wynn TA, Vannella KM (2016). Macrophages in Tissue Repair, Regeneration, and Fibrosis. Immunity.

[31] Jun JI, Lau LF (2018). Resolution of organ fibrosis. J Clin Invest.

[32] Bochaton-Piallat ML, Gabbiani G, Hinz B (2016). The myofibroblast in wound healing and fibrosis: answered and unanswered questions. F1000Res.

[33] Eming SA, Martin P, Tomic-Canic M (2014). Wound repair and regeneration: mechanisms, signaling, and translation. Sci Transl Med.

[34] Herrera J, Henke CA, Bitterman PB (2018). Extracellular matrix as a driver of progressive fibrosis. J Clin Invest.

[35] Choi SY, Bae H, Jeong SH, Park I, Cho H, Hong SP, Lee DH, Lee CK, Park JS, Suh SH, Choi J, Yang MJ, Jang JY, Onder L, Moon JH, Jeong HS, Adams RH, Kim JM, Ludewig B, Song JH, Lim DS, Koh GY (2020). YAP/TAZ direct commitment and maturation of lymph node fibroblastic reticular cells. Nat Commun.

[36] Kostallari E, Valainathan S, Biquard L, Shah VH, Rautou PE (2021). Role of extracellular vesicles in liver diseases and their therapeutic potential. Adv Drug Deliv Rev.

[37] Durant F, Whited JL (2021). Finding Solutions for Fibrosis: Understanding the Innate Mechanisms Used by Super-Regenerator Vertebrates to Combat Scarring. Adv Sci (Weinh).

[38] Henderson J, O'Reilly S (2021). The emerging role of metabolism in fibrosis. Trends Endocrinol Metab.

[39] Wang Z, Chen L, Huang Y, Luo M, Wang H, Jiang Z, Zheng J, Yang Z, Chen Z, Zhang C, Long L, Wang Y, Li X, Liao F, Gan Y, Luo P, Liu Y, Wang Y, XuTan, Zhou Z, Zhang A, Shi C (2021). Pharmaceutical targeting of succinate dehydrogenase in fibroblasts controls bleomycin-induced lung fibrosis. Redox Biol.

[40] Du X, Wu L, Yan H, Jiang Z, Li S, Li W, Bai Y, Wang H, Cheng Z, Kong D, Wang L, Zhu M (2021). Microchannelled alkylated chitosan sponge to treat noncompressible hemorrhages and facilitate wound healing. Nat Commun.

[41] Sobecki M, Krzywinska E, Nagarajan S, Audige A, Huynh K, Zacharjasz J, Debbache J, Kerdiles Y, Gotthardt D, Takeda N, Fandrey J, Sommer L, Sexl V, Stockmann C (2021). NK cells in hypoxic skin mediate a trade-off between wound healing and antibacterial defence. Nat Commun.

[42] Zhang X, Zhang J, Liu Y, Zhu D, Chen D, Zhang Z, Sun Y (2021). Pirfenidone inhibits fibroblast proliferation, migration or adhesion and reduces epidural fibrosis in rats via the PI3K/AKT signaling pathway. Biochem Biophys Res Commun.

[43] Chen K, Yu B, Liao J (2021). LncRNA SOX2OT alleviates mesangial cell proliferation and fibrosis in diabetic nephropathy via Akt/mTOR-mediated autophagy. Mol Med.

[44] Liu P, Luo G, Dodson M, Schmidlin CJ, Wei Y, Kerimoglu B, Ooi A, Chapman E, Garcia JG, Zhang DD (2021). The NRF2-LOC344887 signaling axis suppresses pulmonary fibrosis. Redox Biol.

[45] Tang R, Wang YC, Mei X, Shi N, Sun C, Ran R, Zhang G, Li W, Staveley-O'Carroll KF, Li G, Chen SY (2020). LncRNA GAS5 attenuates fibroblast activation through inhibiting Smad3 signaling. Am J Physiol Cell Physiol.

[46] Li M, Qi C, Song R, Xiong C, Zhong X, Song Z, Ning Z, Song X (2021). Inhibition of Long Noncoding RNA SNHG20 Improves Angiotensin II-Induced Cardiac Fibrosis and Hypertrophy by Regulating the MicroRNA 335/Galectin-3 Axis. Mol Cell Biol.

[47] Yao J, Lin C, Jiang J, Zhang X, Li F, Liu T, Diao H (2021). lncRNA-HEIM Facilitated Liver Fibrosis by Up-Regulating TGF-beta Expression in Long-Term Outcome of Chronic Hepatitis B. Front Immunol.

[48] Lin J, Yang X, Liu S, Luo Z, Chen Q, Sun Y, Ding Z, Chen J (2021). Long non-coding RNA MFAT1 promotes skeletal muscle fibrosis by modulating the miR-135a-5p-Tgfbr2/Smad4 axis as a ceRNA. J Cell Mol Med.

[49] Yang L, Tang J, Chen H, Ge D, Sui T, Que J, Cao X, Ge Y (2016). Taurine Reduced Epidural Fibrosis in Rat Models after Laminectomy via Downregulating EGR1. Cell Physiol Biochem.

[50] Gurtner GC, Werner S, Barrandon Y, Longaker MT (2008). Wound repair and regeneration. Nature.

[51] Stramer BM, Mori R, Martin P (2007). The inflammation-fibrosis link? A Jekyll and Hyde role for blood cells during wound repair. J Invest Dermatol.

[52] Opalenik SR, Davidson JM (2005). Fibroblast differentiation of bone marrow-derived cells during wound repair. Faseb J.

